# Arterial stiffness in hyperthyroid patients is deteriorated due to thyroid hormones

**DOI:** 10.20945/2359-3997000000135

**Published:** 2019-04-26

**Authors:** Canan Yildiz, Mustafa Altay, Sedat Yildiz, Yavuz Çağir, Tolga Akkan, Yasemin Aydoğan Ünsal, Esin Beyan

**Affiliations:** 1 Didim State Hospital Aydin Turkey Didim State Hospital , Aydin , Turkey; 2 Department of Endocrinology and Metabolism Sağlik Bilimleri Üniversitesi Ankara Turkey Department of Endocrinology and Metabolism , Sağlik Bilimleri Üniversitesi (University of Health Sciences) Keçiören SUAM, Ankara , Turkey; 3 Söke Fehime Faik Kocagöz State Hospital Aydin Turkey Söke Fehime Faik Kocagöz State Hospital , Aydin , Turkey; 4 Halil Şivgın Çubuk State Hospital Ankara Turkey Halil Şivgın Çubuk State Hospital , Ankara , Turkey; 5 Çaldiran District State Hospital Van Turkey Çaldiran District State Hospital , Van , Turkey; 6 Department of Internal Medicine Sağlik Bilimleri Üniversitesi Ankara Turkey Department of Internal Medicine , Sağlik Bilimleri Üniversitesi (University of Health Sciences) Keçiören SUAM, Ankara , Turkey

**Keywords:** Arterial stiffness, augmentation index, hyperthyroidism, pulse wave analysis

## Abstract

**Objective:**

The aim of this study is to evaluate and compare arterial stiffness, which is an independent risk indicator for cardiovascular diseases (CVDs), between patients with overt hyperthyroidism, subclinical hyperthyroidism, euthyroidism by antithyroid therapy and healthy volunteers with pulse wave analysis (PWA).

**Subjects and methods:**

A total of 102 volunteers were included in the study (30 in the overt hyperthyroid group, 28 in the subclinical hyperthyroid group and 14 with euthyroidism by antithyroid therapy and 30 healthy). The arterial stiffness measurements of the participants in the study were performed with the Mobil-O-Graph PWA device (I.E.M. GmBH, Stolberg, Germany), which makes cuff based oscillometric measurement from the brachial artery.

**Results:**

Systolic blood pressure, pulse rate, central systolic blood pressure, cardiac output, heart rate-corrected augmentation index (Aix@75) and pulse wave velocity (PWV) measurements were significantly higher in the hyperthyroid group than in the control group. The heart rate and PWV in the subclinical hyperthyroid group were significantly higher than the control group. In the euthyroid group, systolic blood pressure, central systolic blood pressure, cardiac output, cardiac index and PWV were found significantly higher than the control group. There was also a negative correlation between Aix@75 and thyroid-stimulating hormone (TSH), and a positive correlation between Aix@75 and free thyroid hormones.

**Conclusion:**

In our study, we observed that the arterial stiffness was adversely affected by an overt or subclinical increase in thyroid hormones and this correlated with thyroid hormone levels. We recommend that PWV measurement, which is a simple method for detecting CVD risk, can be used in these patients.

## INTRODUCTION

The endocrine system has a wide effect on the cardiovascular system. In particular, the thyroid gland has a specific relationship with cardiovascular system physiology and pathology. Biologically active T3 affects cardiac contractility, heart rate, diastolic function and systemic vascular resistance through genomic and non-genomic effects. Thyroid hormones have the effect of reducing vascular tone on the vascular system and causing normal arteriolar remodeling to be carried out (1). In addition to the direct effects, the thyroid hormones also have indirect effects on cardiovascular function mediated by the autonomic nervous system, renin-angiotensin-aldosterone system, vascular compliance, vasoreactivity and renal function. As with many other systems, hyperthyroidism also reveals some clinical signs in the cardiovascular system. Some of these are systolic hypertension, increased left ventricular mass, exercise intolerance, angina pectoris, and systolic murmur (2).

Many invasive, noninvasive methods and scoring systems are currently used for predicting CVDs. Arterial stiffness is also considered an independent risk indicator for cardiovascular diseases (3-5). Increased stiffness in central arteries leads to undesired hemodynamic consequences such as the expansion of pulse pressure (PP) and increased flow to microcirculation. When the deleterious effects of these changes are evaluated, arterial stiffness can be considered as a predictor of cardiovascular risks (6). Numerous studies involving disease-specific and population-based cohorts have demonstrated that higher carotid-femoral pulse wave velocity (cfPWV) is associated with increased risk for primary and recurrent CVDs (7,8). This data supports the assessment of arterial stiffness as an independent CVD risk factor.

Especially non-invasive approaches in the measurement of arterial stiffness have recently become the foreground. This non-invasive method is called PWA because the calculations are based on the movements of the peripheral pulse waves. PWA is a proven, practical method of detecting arterial stiffness indirectly (9,10). Relatively small number of studies have examined the relationship between thyroid hormones and arterial stiffness, and controversial results have been obtained in these studies.

In the light of all this information, our aim is to evaluate and compare arterial stiffness, which is an independent risk indicator for CVDs, between patients with overt hyperthyroidism, subclinical hyperthyroidism, euthyroidism by antithyroid therapy and healthy volunteers, with pulse wave analysis (PWA).

## SUBJECTS AND METHODS

This study was approved by the Research Ethics Committee of the University of Health Sciences, Keçiören SUAM (date: 02/02/2016 and reference number 126). All the methods used in the study were carried out in accordance with the approved guidelines and Declaration of Helsinki, Ethical Principles for Medical Research Involving Human Subjects. Patients who have been diagnosed with overt or subclinical hyperthyroidism, patients with euthyroidism who were previously diagnosed with hyperthyroidism and euthyroid healthy volunteers without systemic disease referred to our outpatient clinics for Endocrinology and Metabolic Diseases between 01/06/2016 and 01/12/2016 were included. Written informed consent was obtained from all individual participants included in the study. The inclusion criteria for overt and subclinical hyperthyroidism groups were 18 years old or over, not receiving anti-thyroid or levothyroxine therapy in the last 1 year. The inclusion criteria for the euthyroid group were 18 years old or over, being taken antithyroid therapy due to hyperthyroidism and being in the euthyroid state at least 3 months. Exclusion criteria for all groups were defined as the presence of chronic diseases (diabetes mellitus, hypertension, coronary artery disease, chronic kidney disease, subclinical or obvious hypothyroidism), pregnancy and lactation for all groups. Detailed medical histories of all participants were obtained, and physical examination of all participants were performed. The age and gender of these patients were learned and height and weight measurements were made. The body mass index (BMI) was calculated by the weight (kg)/height (m) ^2^ formula.

Blood samples were taken in the morning hours after at least 10 hours of fasting. Free T3, free T4 and TSH levels were studied by chemiluminescence method with Abbott Architect i2000 device (Illinois, USA). It was defined as euthyroidism if the levels of TSH (0.35-4 mIU/mL), fT3 (1.71-4.71 pg/mL) and fT4 (0.8-1.9 ng/mL) were within the normal reference limits. Overt hyperthyroidism was defined as low TSH level with elevated free T3 and free T4 concentrations. Subclinical hyperthyroidism was defined as low TSH level with normal free T3 and free T4 concentrations. TSH Receptor Antibody (TRAB) measurements were performed by Beckman Coulter Immunotech device (Florida, USA) with immunoassay method. anti-thyroglobulin (Anti-TG) and anti-thyroid peroxidase (Anti-TPO) measurements were studied by chemiluminescence method with Abbott Architect i2000 device (Illinois, USA). These biochemical tests were performed for etiology in cases of overt and subclinical hyperthyroidism.

### Evaluation of pulse wave analysis and arterial stiffness

In this study, a PWA device, Mobil-O-Graph PWA/ABPM (I.E.M. GmBH, Stolberg, Germany), which performs cuff based oscillometric measurement from the brachial artery, was used to collect data. The Mobile-O-Graph PWA device is a blood pressure measurement device approved by the British Hypertension Society (BHS) and the European Society of Hypertension (ESH). The measurement reliability of this device has been demonstrated by comparing data obtained by invasive catheterization, magnetic resonance imaging and tonometry devices (11).

Participants were kept sitting in a quiet room for 15 minutes before measuring arterial stiffness. Suitable cuffs were selected for the arms of the participants. The cuff was placed just above the right arm elbow. Pulse wave velocity analysis was performed from the brachial artery 4 times at 5-minute intervals at the sitting position. A bluetooth connection was established between the device and the software which is developed to monitor the device’s data. Age, gender, height and weight information of all participants were entered into the software program. Firstly, blood pressure measurements were made with the device and the pressure, where the brachial artery was fully compressed, was determined. After 30 seconds of recovery, the brachial artery was completely re-compressed. When pulse waves reached the point where there is no flow, it is detected and magnified by special sensors in the device. So that the peripheral pulse wave was obtained. The data obtained here is classified as very high quality and low quality by the software. Only very high-quality data were evaluated for the study.

The central aortic pulse wave was calculated from measured brachial artery pulse wave by special software. The calculated PWA was completed with aortic pulse wave. As a result of these measurements; systolic blood pressure (SBP), diastolic blood pressure (DBP), mean blood pressure (MBP), heart rate (HR), PP, central SBP, central DBP, central PP, cardiac output (CO), peripheral vascular resistance, cardiac index, body surface, augmentation pressure (AP), augmentation index corrected based on pulse rate 75 beats/min (Aix@75), reflectance magnitude and PWV data were obtained. The obtained data was transferred to the software program and saved.

### Statistical analysis

SPSS 22.0 program was used for statistical analysis of the data. Statistically, p < 0.05 was considered significant. Descriptive statistics of patients and control groups were performed. Categorical values were reported by number and percentage. The Kolmogorov-Smirnov/Shapiro-Wilk’s test and histogram graphs of data were used to assess whether the data showed a normal distribution. Data that do not correspond to normal distribution are expressed as median and minimum-maximum values and the nonparametric analysis was performed. Mann-Whitney U test was used to evaluate countable data with non-normal distribution. The Kruskal-Wallis test was performed to compere biochemical values and PWA parameters among groups. Post hoc analysis was performed and Bonferroni correction was made. The Chi-square test was used to compare the categorical variables. While investigating the associations between non-normally distributed and/or ordinal variables, the correlation coefficients and their significance were calculated using the Spearman correlation test.

## RESULTS

Thirty patients were included for each overt hyperthyroidism, subclinical hyperthyroidism and euthyroidism groups. Also, 30 volunteers were included in the control group. Patients who were diagnosed with high blood pressure and high blood glucose levels were excluded from the study even though they were not diagnosed during the initial evaluation. In conclusion, we continued to work with 30 patients in the overt hyperthyroid group, 28 in the subclinical hyperthyroid group and 14 in the euthyroid group and 30 healthy volunteers. Thyroid hormone levels and diagnoses of the patients participating in the study are given in Table 1.

**Table 1:** The median values of thyroid hormone levels and causes of hyperthyroidism among the groups

		Overt Hyperthyroidism (n = 30)	Subclinical Hyperthyroidism (n = 28)	Euthyroidism (n = 14)	Control (n = 30)
TSH (mIU/mL)	< 0.01(< 0.01-0.02)		0.17 (0.00-0.49)	1.35 (0.53-3.10)	1.51 (055-3.60)
fT4 (ng/mL)	2.17 (1.46-4.27)		1.13 (0.84-1.70)	1.13 (0.87-1.25)	1.01 (0.83-1.35)
fT3 (ng/mL)	7.45 (4.41-30)		3.18 (2.36-4.30)	2.88 (2.42-3.39)	2.92 (0.83-3.32)
Graves’ disease (n)	19		7	3	-
Toxic adenoma (n)	-		7	1	-
TMNG (n)	-		7	7	-
Others (n)	11		7	3	-

Others: subclinical granulomatous thyroiditis, Hashitoxicosis, postpartum thyroiditis, subacute lymphocytic thyroiditis. TSH: thyroid stimulating hormone; fT4: thyroxine; fT3: triiodothyronine; TRAB: TSH receptor antibody; Anti-TG: anti-thyroglobulin; Anti-TPO: anti-thyroid peroxidase; TMNG: toxic multinodular goiter.

The data in parentheses show the minimum and maximum value range.

Control and patient groups were similar in terms of age, sex, and BMI (Table 2). SBP (p = 0.02), HR (p < 0.01), central SBP (p = 0.04), CO (p = 0.02), Aix@75 (p < 0.01), PWV (p < 0.01) showed the significant differences between the groups (Table 2). Other data were not statistically different between the groups.

**Table 2:** Demographic characteristics and PWA results (median values) in the study groups

	Overt Hyperthyroidism (n = 30)	Subclinical Hyperthyroidism (n = 28)	Euthyroidism (n = 14)	Control (n = 30)	p
Female gender n (%)	22 (73.3%)	25 (89.3%)	12 (85.7%)	22 (73.3%)	NS
Age	32.5 (19-65)	35 (20-66)	41.87 (23-69)	31 (19-64)	NS
BMI – kg/m ^2^	23.7 (15.7-40)	26.9 (17.3-35)	26.45 (18.7-35.6)	23.8 (18.3-43.8)	NS
SBP – mmHg	120 (102-140)	115.5 (93-140)	125 (110-139)	114.5 (101-138)	**0.02** ^**a**^
DBP – mmHg	73 (54-90)	71 (52-87)	75.5 (58-90)	69 (49-90)	NS
MBP – mmHg	93.5 (78-119)	92.5 (70-116)	99 (82-111)	90 (73-112)	NS
HR – beats/min	98.5 (70-117)	87 (56-110)	77 (50-91)	75 (53-96)	**< 0.01** ^**b,c,d**^
PP – mmHg	46.5 (33-73)	45 (30-67)	49.5 (43-59)	44.5 (30-63)	NS
cSBP – mmHg	108 (88-134)	107 (85-138)	116.5 (104-130)	104.5 (92-132)	**0.04a**
cDBP – mmHg	75 (57-90)	72.5 (53-88)	77.5 (59-90)	71.5 (50-90)	NS
sPP – mmHg	34 (23-51)	35 (22-50)	41 (32-57)	36.5 (21-48)	NS
CO – lt/min	4.85 (4.20-45)	4.70 (3.40-6.40)	5.40 (4.50-48)	4.65 (3.30-6.10)	**0.02** ^**a**^
PR –smmHg/mL	1.18 (0.95-1.43)	1.17 (0.92-1.70)	1.15 (0.80-1.31)	1.20 (0.80-1.58)	NS
CI – lt/min/m ^2^	2.80 (2.40-3.80)	2.85 (2.30-4.00)	2.95 (2.60-3.90)	2.70 (1.70-3.60)	NS
AP – mmHg	9 (2-19)	8 (3-26)	8 (4-21)	7 (2-19)	NS
Aix@75 (%)	35.5 (10-58)	29 (5-45)	20 (8-45)	25 (9-41)	**< 0.01** ^**c,d**^
PWV – m/s	5.6 (4.8-9.7)	5.7 (4.5-10.3)	6.35 (4.8-10.1)	5.2 (4.5-8.5)	**< 0.01** ^**a,b**^

NS: not significant; BMI: body mass index. SBP: systolic blood pressure; DBP: diastolic blood pressure; MBP: mean blood pressure; PP: pulse pressure; cSBP: central systolic blood pressure; cDBP: central diastolic blood pressure; CO: cardiac output; PR: peripheral resistance; CI: cardiac index; AP: augmentation pressure; Aix@75: augmentation index (corrected based on pulse rate 75 beats/min); PWV: pulse wave velocity.

a: between euthyroidism and control groups adjusted p < 0.05.

b: between subclinical hyperthyroidism and control groups adjusted p < 0.05.

c: between overt hyperthyroidism and control groups adjusted p < 0.05.

d: between euthyroidism and overt hyperthyroidism groups adjusted p < 0.05.

SBP, HR, central SBP, CO, Aix@75 and PWV measurements were significantly higher in the hyperthyroid group than in the control group. HR and PWV measurements were significantly higher in the subclinical hyperthyroid group than in the control group. In the euthyroid group, SBP, central SBP, CO and PWV measurements were significantly higher than the control group (Table 2).

**Figure 1:**
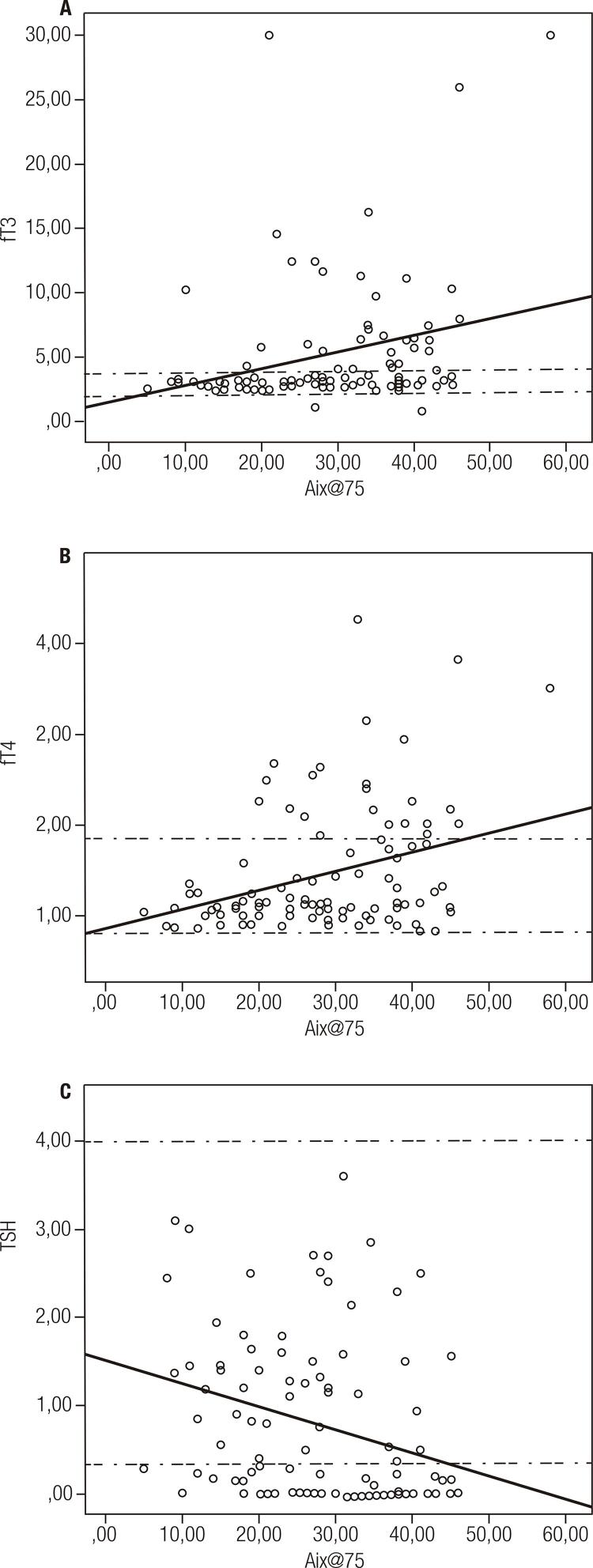
Correlation analysis between TSH (0.35-4 mIU/mL), fT4 (0.8-1.9 ng/mL) and fT3 (1.71-4.71 pg/mL) with Augmentation Index. “--” shows the reference values a. Correlation between sT3 and Aix@75 p < 0.001 r = 0.335. b. Correlation between sT4 and Aix@75 p = 0.004 r = 0.282. c. Correlation between TSH and Aix@75 p < 0.001 r = -0.363.

Correlation of thyroid function tests and thyroid autoantibodies with PWA parameters was assessed. As a result of the analysis, negative correlation was found between Aix@75 and TSH (p < 0.001, r = -0.363); on the other hand, there was positive correlation between Aix@75 and fT3 and fT4 (relatively p = 0.004, r = 0.282 and p = 0.001, r = 0.335) (Figure 1).

## DISCUSSION

In our study, we found that there was an increase in the arterial stiffness and a significant correlation between Aix@75, the arterial stiffness parameter, and thyroid hormone levels.

The relationship between thyroid hormone levels and arterial stiffness has become more current in recent years and has been investigated in some community-based studies. For example; In the 10,027-person study by Wang and cols., the PWV measurements of 812 individuals were analyzed by an oscillometric method. Unlike similar studies, a negative correlation between PWV and free T4 was observed; but there was no correlation with TSH and free T3. It was noted that the augmentation index was not examined in the study and the cross-sectional nature of the study was the weaknesses of the study. In the population-based SardiNIA study, PWV and augmentation index measurements were performed by oscillometric method in participants, and as a result, the linear positive correlation between free T4 and PWV was observed. There was no significant correlation with TSH and free T3. It was reported that the increase in fT4 contributes to the increase in PWV, as well as the aging of the vascular system. Another point that was mentioned in the study was the relationship between hypothyroidism and increased arterial stiffness, which was reported in earlier studies, connected to dyslipidemia (12).

Obuobie and cols., a first study of the relationship between thyrotoxicosis and arterial stiffness, found lower augmentation index in a small number (n = 20) of thyrotoxicosis cases than the control group during the diagnosis (13). At 6 months after treatment, when the cases were euthyroid, similar data were obtained compared to the control group. In this study, it was reported that thyrotoxicosis reduces this risk by decreasing arterial stiffness as a counter-mechanism to PP and that there was no cardiovascular risk in thyrotoxicosis (14). However, the low number of patients participating in the study, the high average age (mean age 48) and the absence of thyrotoxicosis causes were important limitations of the study.

Moulakakis and cols. reported that rats with thyrotoxicosis had a significant arterial stiffness in the aortic wall. An increase in internal and external diameter, smooth muscle cell and collagen density was observed in the aortic wall. On the other hand, a reduction in elastin lamina thickness and elastin density was found.

It was reported that arterial stiffness resulting from thyrotoxicosis might have been associated with increased arterial pressure, which increased the risk of developing vascular complications. Despite low lipid levels, an increase in arterial stiffness was found in the study (15). In another study, ambulatory arterial stiffness indices (AASI) and blood pressure changes were assessed using data obtained from 23 participants with overt hyperthyroidism, 36 participants with subclinical hyperthyroidism and 25 healthy volunteers by following 24-hour ambulatory blood pressure monitoring. In this study, systolic and DBP were significantly higher in the hyperthyroid group compared to the other groups. However, AASI results did not show any significant difference between the groups. When all groups were evaluated, the positive correlation between AASI and free thyroid hormone levels was observed but no correlation with TSH was detected. Similarly, in our study, systolic and DBP were higher in the overt and subclinical hyperthyroid groups than the control group. However, unlike this study, Aix@75 and PWV measurements were significantly higher in both the overt and subclinical hyperthyroid groups. In addition, the positive correlation between Aix@75 and free thyroid hormone levels, and the negative correlation between Aix@75 and TSH were observed.

In our study, the increase in SBP, HR and CO, especially in the overt hyperthyroid group, can be explained by the known and common effects of thyroid hormones on the cardiovascular system. In the subclinical hyperthyroidism group, the increase in these parameters was not as noticeable as in the overt hyperthyroidism group. This situation may be explained by the lower peripheral thyroid hormone levels. In the euthyroid group SBP, central SBP, CO and PWV were also higher than the control group.

There were some major differences when focusing on PWV and Aix@75 (Table 2). PWV was higher especially in the euthyroidism and the subclinical hyperthyroidism groups compared to the control group. On the other hand, Aix@75 level was higher in the overt hyperthyroidism group. This is an issue that needs to be considered in detail. In the euthyroidism and the subclinical hyperthyroidism groups, until the treatment, the cardiovascular system may be exposed to thyroid hormones, even if small amounts, and prolonged exposure to the thyroid hormones may affect PWV by creating more lasting effects on vascular structures. On the other hand, in the overt hyperthyroidism group, because of its high hormone effect, clinical symptoms occur in a much shorter time. There is not enough time for the changes in vascular structures which cause the increase in PWV. However, short-term high-dose hormone exposure may affect Aix@75 but this exposure time may not be enough to make significant changes in PWV, because of early treatment. These are very recent data and there is no previous study on this topic. With more extensive study, new information can be obtained.

Focusing on PWV and Aix@75, a comparative study of efficacy between PWV and Aix@75 for the efficacy of arterial stiffness has not been conducted but it is inferred from the data that Aix@75 is more susceptible to arterial stiffness. This is because of the augmentation index, corrected based on pulse rate 75 beats/min, increases the strength and significance of the obtained data, especially considering the pulse rate increase in thyrotoxicosis studies may lead to wrong evaluations. In addition, a more detailed evaluation of Aix@75 has shown a close relationship between Aix@75 and thyroid hormones. This may explain that Aix@75 is only significant the overt hyperthyroidism group when compared to the control group. A negative correlation between Aix@75 and TSH in the correlation analysis also reinforces our findings. In previous studies, correlation evaluations were made between TSH and PWV and augmentation index, but no significant correlation could be shown. It is important that this is shown in this study.

It is known that arterial stiffness is evaluated as a predictive parameter for cardiovascular risks (6). On the other hand, in overt hyperthyroidism, high thyroid hormone levels have many effects on the cardiovascular system and overt hyperthyroidism is a risk factor for CVDs. In addition to overt hyperthyroidism, subclinical hyperthyroidism also similarly increases the risk of atrial fibrillation and CVDs in a similar way (16). In our study, the relationship between Aix@75 and thyroid hormone levels were emphasized, indicating that these two conditions are related to each other. In current approaches, the decision to treat subclinical hyperthyroidism is based on the additional cardiovascular risk profile of the patients. Assessment of cardiovascular risk in these patient groups with the evaluation of arterial stiffness may be a new and reliable approach in establishing treatment indications. However, there is a need for more extensive and greater studies for this approach.

There were some limitations in the study. The first of these; when the euthyroid group was being formed; patients who had previously been treated with hyperthyroidism and became euthyroid were included. The number of these patients was relatively low due to the detection of exclusionary conditions for the study. Second, the diagnosis of dyslipidemia in participants was excluded by the anamnesis. Serum lipid levels of patients were not measured. One of the strengths of the study was the assessment of euthyroid cases with previous hyperthyroidism history, subclinical hyperthyroidism and the overt hyperthyroidism in a single study. Another was that there was no difference in age, gender, and BMI among all groups and those other parameters that might affect arterial stiffness were excluded.

As a result; in our study, we found that the overt or subclinical increase in the thyroid hormone levels adversely affected arterial stiffness which is a reliable risk indicator for CVDs. In the case of hyperthyroidism, especially PWV and Aix@75 are significantly altered. From this point of view, patients with thyrotoxicosis, even if taken high doses of thyroid hormone therapy, should be evaluated and informed previously in terms of cardiovascular risks. We hope that our research will lead to more comprehensive and further studies.
